# Self-reported depression is increasing among socio-economically disadvantaged adolescents – repeated cross-sectional surveys from Finland from 2000 to 2011

**DOI:** 10.1186/1471-2458-14-408

**Published:** 2014-04-28

**Authors:** Antti Torikka, Riittakerttu Kaltiala-Heino, Arja Rimpelä, Mauri Marttunen, Tiina Luukkaala, Matti Rimpelä

**Affiliations:** 1 Kanta-Häme Central Hospital, 13530 Hämeenlinna, Finland; 2 Department of Adolescent Psychiatry, Tampere University Hospital, P.O. Box 2000, FI-33521 Tampere, Finland; 3 School of Medicine, University of Tampere, Tampere, Finland; 4 School of Health Sciences, University of Tampere, FIN- 33014 Tampere University, Tampere, Finland; 5 Department of Mental Health and Substance Abuse Services, National Institute for Health and Welfare, P.O. Box 30, FIN-00271 Helsinki, Finland; 6 Department of Adolescent Psychiatry, University of Helsinki and Helsinki University Central Hospital Helsinki, Tampere, Finland; 7 Science Center, Pirkanmaa Hospital District, P.O. Box 2000, 33521 Tampere, Finland

**Keywords:** Depression, Adolescence, Epidemiology, Time trends, Health inequality

## Abstract

**Background:**

Adolescent depression is more common in lower socio-economic groups. Whether this pattern has changed over time, is not known.

We examined the prevalence of self-reported depression and its changes in socio-economic groups from 2000 to 2011 among Finnish adolescents.

**Methods:**

Data were based on classroom surveys every second year from 2000–2001 to 2010–2011 using nationwide samples of 14–16-year old Finns (n = 618,084). Data were collected using self-administered questionnaires including questions on health, health behaviours, and school experiences. Depression was measured with a Finnish modification of the 13-item Beck Depression Inventory, and divided into no, mild, moderate and severe depression. The association between depression and the social background (parents’ education and employment) over time was studied using a multinomial regression analysis.

**Results:**

The prevalence of self-reported severe depression slightly increased from 2000–2001 to 2010–2011 in girls. In boys a slight increase was observed when adjusting for background variables. The differences in the prevalence of depression between the social background groups persisted over the entire study period. In both sexes, severe depression nearly doubled among those adolescents whose parents were unemployed and had a low education level; among boys, the prevalence was 6.5% in 2000–2001 and 12.8% in 2010–2011, and among girls 6.4% and 11.4% respectively.

**Conclusion:**

The largest increases in prevalence of severe depression are seen among socio-economically disadvantaged adolescents. This suggests that inequalities in mental health may become an increasing concern.

## Background

Depression is a common mental disorder characterized by a depressed mood or a loss of interest or pleasure in daily activities for more than two weeks [1] and a variety of additional symptoms, which may occur. A diagnosis of depression requires clinical assessment, but in epidemiological research, depression is often measured with questionnaires quantifying and summarizing the different symptoms of depression. Depression is then defined as scoring above a pre-defined cut-off score. A variety of expressions are used for depression measured by self-report questionnaires, often interchangeably. In this article, when referring to previous research, we use the term used by the original authors, and as to our own research, we use term “self-reported depression” as referring to those who scored above cut-point for depression in our data.

The prevalence of depression increases significantly during the transition from childhood to adolescence [2-5], with the highest incidence at ages 15 to18 [6]. The point prevalence of adolescent depressive disorders ranges from 1.5% to 8% in community samples, and lifetime prevalence estimates through adolescence are as high as 20% [7-9]. The equal sex ratio in early childhood changes to a female preponderance in adolescence, when depression is approximately twice as common among girls [2,4,10,11]. Whether adolescent depression has increased over time, remains controversial. A meta-analysis by Costello et al. [12] and a review by Richter et al. [13] concluded that there is no evidence of an increase in depressive disorders over the past 30 years. Some studies [4,14,15], however, suggest an increase in prevalence and a decrease in age at the onset of depression. This has been particularly evident in those studies that extended to the middle of the first decade beyond the year 2000. In Iceland depressive symptoms increased significantly among girls from 1997 to 2006 and the proportion of adolescents visiting mental health services also increased [16]. In Great Britain twice as many adolescents reported frequent feelings of depression in 2006 compared to 1986 [17]. In Finland 8-year-old girls exhibited an increase in depressive symptoms from 1989 to 2005 [18].

Low family income and socio-economic status [19,20], as well as exposure to poverty in the early stages of life are known risk factors for adolescent depression [20]. Furthermore, limited material resources in a family predict a decreased health- related quality of life in adolescence, and parental educational level has an impact on psychological wellbeing, moods, and emotions [21]. Previous studies on time trends in depression have not considered the possibility that changes in the prevalence of depression among adolescents may vary over population groups. Societal changes may affect population groups differently: for example, vulnerable groups are at higher risk during a period of economic recession.

The risk of poverty and exclusion from the labour market and education is often highest among the same population groups [22]. The proportion of families with children living in poverty has increased in the 2000s [23]. Earnings among the bottom 10 per cent of the population have increased at a slower rate than in households on average, and the number of families living below the poverty line has doubled since 1995 [22].

Depressive symptoms are suggested to be on a continuum, while the diagnosis of depression is categorical [24]. Minor changes in case definition or in measuring instruments may yield major differences in prevalence estimates [25]. A problem in many previous time trend studies on adolescent depression is that they have not used comparable samples or comparable measurement instruments. The School Health Promotion Study in Finland provides an excellent opportunity to study time trends among 14–16-year-olds with large nationwide samples, using the same measurement instrument and the same collection method throughout the study.

The present study examines changes in self-reported adolescent depression from 2000–2001 to 2010–2011 using the national data of the School Health Promotion Study. Furthermore, we study whether changes over time vary according to the socio-economic background of the family in terms of parental unemployment and education.

## Methods

The School Health Promotion Study of the National Institute for Health and Welfare is a school- based survey designed to examine the health, health behaviours, and school experiences of Finnish teenagers. The survey is conducted among 8th and 9th graders biennially in the same regions in Finland with the pooled 2-year data (2000–2001, 2002–2003, 2004–2005, 2006–2007, 2008–2009, and 2010–2011). Participants completed the questionnaire anonymously during a school lesson under the supervision of a teacher, who did not interfere with the responses. Participants were informed about the nature of the study as well as the voluntary nature of participation in both oral and written form, and returning the survey was considered consent to participate. The questionnaire took 30 to 45 minutes to complete and was then placed in an envelope, sealed, and returned directly to the research centre. The study was approved by the ethics committee of Pirkanmaa Hospital District.

### Sample and participants

The survey was sent to every municipality in Finland, which decided if the schools in their area would participate in the survey. The number of schools participating in the survey ranged from 578 to 831 biennially. Our data include those 535 schools that participated in all six of the surveys. Altogether, 618,084 (94,635–108,320 biennially) pupils were present on the survey days and returned the questionnaire in these schools. Approximately 10% to 15% of pupils were absent from school due to illness or other reasons. Subjects (<0.7%) with incomplete responses on the depression rating scale were excluded from the analyses. The timing of the study, and sample and data collection methods were held constant in each survey.

### Measures

Depression was measured by a 12-item version of the R-BDI [26], a modification of the 13-item Beck Depression Inventory (BDI) [27,28] validated in Finnish [26,29]. In the R-BDI, an introductory question and one positive choice answer were added for each item. Thus, the R-BDI constructs a dimensional continuum in which positive mood and depressive symptoms are the two end points of the continuum [29,30]. The reliability and validity of the BDI [31] and the Finnish modification R-BDI [26,29] are well established in both adult and adolescent samples. The psychometric properties of the scale have been shown to be good in the School Health Promotion Study [32]. The R-BDI comprises statements describing an increasing intensity of depressive emotions and cognitions, scoring 0–3 each. Scores of 0–4 are classified as no depression, 5–7 as mild, 8–15 as moderate, and 16 and over as severe depression [27]. We used a 12-item version that omitted the item eliciting suicidal ideation, because in 1998 the Ministry of Education disapproved of including this item in a school survey, fearing that asking about suicidality might provoke it. We previously demonstrated that the 12-item version is best used with the same cut-off points as the original 13-item BDI [33].

The socio-economic variables recorded were: sex, parental education level, unemployment in the family during the past 12 months, and family structure. Family structure was taken into account for the analyses because it has a known association with adolescent depression [34]. Parental education level was categorised as low (basic only), medium (vocational school), or high (university level/academic) based on the parent with the highest level of education. Parental unemployment was elicited as follows: “Have your parents been unemployed or been laid off work during the past 12 months”. The response alternatives were: “none/one of the parents/both parents”. Unemployment in the family was dichotomised as none versus one parent/both parents, and family structure as living with both parents versus other.

### Statistical analysis

Distributions of depression and socioeconomic variables among girls and boys during the time period 2000–2011 are presented in Table 1. Distributions, as percentages of depression, were expressed in categories of parent’s education and unemployment in Table 2 separately for girls and boys. Multivariate associations were studied using multinomial logistic regression results shown by odds ratios with 95% confidence intervals. Depression was entered as the dependent variable showing results for severe depression (Beck > 15), moderate depression (Beck 8–15) and mild depression (Beck 5–7) versus no depression (Beck 0–4). Due to the missing values of depression scale, 3823 cases were excluded from the analyses. In the first model (Model 1), categorical time periods (2000–2001, 2002–2003, 2004–2005, 2006–2007, 2008–2009, 2010–2011) were entered as an independent factor, with the period 2000–2001 entered simultaneously as a reference category (Table 3). In the second model (Model 2), grade, family structure (living with both parents/other), unemployment in family during the past 12 months (yes/no) and parental education (low/medium/high) were added to the model as covariates. In addition to this, instead of categorical time periods, time has been modelled also as continuous factor. Finally, in Table 4, interaction of parental education with parental unemployment was modelled as factor with family structure and grade as covariates separately for each time period from 2000–2001 to 2010–2011.

**Table 1:** Distribution of depression and socioeconomic background by study year and sex

	**Boys**						**Girls**					
**Year**	**2000–2001**	**2002–2003**	**2004–2005**	**2006–2007**	**2008–2009**	**2010–2011**	**2000–2001**	**2002–2003**	**2004–2005**	**2006–2007**	**2008–2009**	**2010–2011**
**Number of participants**	**47586**	**50774**	**53057**	**54315**	**54132**	**51116**	**47049**	**49236**	**51713**	**54005**	**54035**	**51066**
	**%**	**%**	**%**	**%**	**%**	**%**	**%**	**%**	**%**	**%**	**%**	**%**
Depression												
No (0–4)	83.7	85.1	84.0	83.1	83.1	84.9	67.5	70.4	68.7	68.3	67.9	68.0
Mild (5–7)	8.0	7.4	7.9	8.4	8.4	7.5	15.0	13.9	14.1	14.2	14.4	14.0
Moderate (8–15)	6.2	5.6	6.0	6.3	6.3	5.5	13.5	12.2	13.0	13.4	13.4	13.4
Severe (>15)	2.1	2.0	2.1	2.2	2.2	2.2	4.0	3.5	4.2	4.1	4.3	4.7
Unemployment in the family during the last year												
No	67.4	71.0	72.4	75.7	73.1	68.8	66.5	69.9	71.0	74.8	72.2	67.7
Yes	29.4	26.6	25.6	22.2	25.0	29.7	31.8	28.8	28.0	24.1	26.6	31.5
Unknown	3.1	2.5	2.0	2.1	1.9	1.5	1.7	1.4	1.1	1.1	1.1	0.9
Parental education												
High	50.5	54.1	60.1	62.0	64.6	59.9	44.6	49.3	55.5	57.7	60.7	60.2
Medium	32.5	32.4	27.5	25.9	23.9	28.1	38.0	37.0	32.6	31.1	28.8	27.1
Low	8.6	7.3	6.5	5.5	4.4	6.6	9.5	7.6	6.7	5.7	4.5	7.8
Unknown	8.3	6.2	5.9	6.5	7.1	5.4	8.0	6.1	5.1	5.5	6.0	4.8

**Table 2:** Depression (%) according to study year and parents’ education and employment status (number of persons in the group presented in the parentheses)

			**Boys**						**Girls**			
	**Depression (%)**											
**Parents’ employment and education**	**2000–2001**	**2002–2003**	**2004–2005**	**2006–2007**	**2008–2009**	**2010–2011**	**2000–2001**	**2002–2003**	**2004–2005**	**2006–2007**	**2008–2009**	**2010–2011**
**High education employed**	(18008)	(21333)	(24822)	(27125)	(27223)	(22366)	(15707)	(18906)	(22083)	(24913)	(25320)	(22227)
Mild	7.2	6.8	7.0	7.5	7.3	6.7	14.2	12.7	13.1	13.0	13.2	12.6
Moderate	5.7	4.7	5.0	5.0	5.2	4.6	10.8	9.7	10.9	11.0	11.1	10.8
Severe	1.6	1.3	1.5	1.4	1.5	1.5	2.7	2.5	2.9	3.1	3.1	3.3
**High education unemployed**	(5957)	(6017)	(6921)	(4402)	(7658)	(8162)	(5208)	(5348)	(6563)	(6204)	(7430)	8459)
Mild	9.7	8.9	9.8	10.1	10.2	8.6	15.5	15.2	15.5	16.1	16.2	16.0
Moderate	8.0	7.0	8.4	9.4	9.1	7.5	15.3	14.2	15.1	17.7	16.0	15.7
Severe	2.9	3.1	2.8	3.6	3.2	2.9	4.8	4.7	5.9	5.4	5.7	5.7
**Medium education employed**	(9765)	(10787)	(9799)	(10077)	(8728)	(9426)	(10998)	(11574)	(11006)	(11651)	(10242)	(8728)
Mild	7.2	6.6	7.4	7.8	7.9	6.7	14.3	13.3	13.8	14.4	14.4	13.6
Moderate	4.9	4.6	5.2	5.5	5.7	4.3	12.4	11.7	12.1	12.9	12.8	12.4
Severe	1.4	1.2	1.3	1.5	1.3	1.3	3.3	3.0	3.6	3.4	3.9	4.0
**Medium education unemployed**	(5659)	(5586)	(4697)	(3978)	(4177)	(4903)	(6829)	(5670)	(5823)	(5105)	(5287)	(5098)
Mild	9.4	8.6	10.1	10.9	11.1	9.2	16.5	16.3	16.6	16.5	16.7	15.5
Moderate	7.4	6.9	7.2	8.5	7.6	7.5	16.8	15.3	17.2	18.0	18.1	17.4
Severe	2.0	2.3	2.7	3.5	3.2	2.3	5.1	4.7	5.7	6.6	6.5	6.8
**Low education employed**	(2503)	(2372)	(2095)	(1907)	(1432)	(1908)	(2566)	(2235)	(2052)	(1956)	(1410)	(2139)
Mild	8.1	7.6	8.0	8.6	8.4	8.9	15.1	13.8	13.6	13.9	15.1	13.8
Moderate	5.6	5.5	5.8	7.4	8.0	5.3	14.1	13.0	14.7	14.3	15.7	14.8
Severe	2.2	2.9	2.7	2.4	4.1	2.7	4.6	3.5	4.4	5.3	5.4	5.1
**Low education unemployed**	(1580)	(1322)	(1319)	(1081)	(911)	(1441)	(1871)	(1493)	(1423)	(1125)	(1003)	(1851)
Mild	9.9	9.5	9.8	10.9	11.1	9.5	16.3	15.6	16.4	16.8	17.4	17.2
Moderate	7.7	10.1	7.5	11.7	11.1	7.2	18.1	18.1	19.2	18.1	18.2	20.3
Severe	6.5	9.1	9.1	13.0	13.7	12.8	6.4	6.8	9.0	9.9	12.4	11.5

**Table 3:** Mild, moderate and severe depression compared to No depression according to study year

**Time period**	**2000–2001**	**2002–2003**	**2004–2005**	**2006–2007**	**2008–2009**	**2010–2011**	**2000–2011**
	**OR (95% CI)**	**OR (95% CI)**	**OR (95% CI)**	**OR (95% CI)**	**OR (95% CI)**	**OR (95% CI)**	**OR (95% CI)**
**Boys**							
Model 1							
Mild	1.00	0.90 (0.86-0.95)	0.99 (0.94-1.03)	1.05 (1.01-1.10)	1.05 (1.01-1.10)	0.92 (0.88-0.96)	1.00 (1.00-1.01)
Moderate	1.00	0.88 (0.84-0.93)	0.96 (0.91-1.01)	1.02 (0.97-1.07)	1.03 (0.98-1.08)	0.87 (0.83-0.92)	1.00 (0.99-1.01)
Severe	1.00	0.95 (0.87-1.04)	1.00 (0.92-1.09)	1.07 (0.98-1.16)	1.08 (0.99-1.18)	1.04 (0.96-1.14)	**1.02** (1.01-1.03)
Model 2							
Mild	1.00	0.91 (0.87-0.96)	1.00 (0.95-1.04)	1.06 (1.02-1.11)	1.06 (1.02-1.11)	0.93 (0.88-0.97)	1.01 (1.00-1.01)
Moderate	1.00	0.89 (0.85-0.94)	0.98 (0.93-1.03)	1.04 (0.99-1.09)	1.05 (1.00-1.10)	0.89 (0.84-0.94)	1.00 (0.99-1.01)
Severe	1.00	0.97 (0.89-1.06)	1.04 (0.95-1.13)	**1.10** (1.01-1.20)	**1.11** (1.02-1.21)	1.08 (0.99-1.18)	**1.03** (1.01-1.04)
N	47586	50774	53057	54315	54132	51116	310980
**Girls**							
Model 1							
Mild	1.00	0.89 (0.86-0.92)	0.93 (0.89-0.96)	0.94 (0.91-0.98)	0.96 (0.92-0.99)	0.93 (0.89-0.96)	1.00 (0.99-1.00)
Moderate	1.00	0.86 (0.83-0.90)	0.95 (0.91-0.98)	0.98 (0.95-1.02)	0.98 (0.95-1.02)	0.98 (0.95-1.02)	**1.01** (1.00-1.02)
Severe	1.00	**0.84** (0.79-0.90)	1.04 (0.97-1.10)	1.02 (0.96-1.08)	**1.08** (1.01-1.15)	**1.16** (1.09-1.24)	**1.04** (1.03-1.06)
Model 2							
Mild	1.00	0.90 (0.86-0.93)	0.94 (0.90-0.97)	0.95 (0.92-0.99)	0.97 (0.93-1.003)	0.94 (0.90-0.97)	1.00 (0.99-1.00)
Moderate	1.00	0.88 (0.84-0.91)	0.97 (0.93-1.01)	1.01 (0.97-1.05)	1.007 (0.97-1.05)	1.009 (0.97-1.05)	**1.02** (1.01-1.02)
Severe	1.00	**0.86** (0.81-0.92)	**1.07** (1.01-1.14)	1.06 (0.99-1.12)	**1.12** (1.05-1.19)	**1.21** (1.14-1.29)	**1.05** (1.04-1.06)
N	47049	49236	51713	54005	54035	51066	307104

Model 1: time period as factor, no covariates.

Model 2: time period as factor; grade, family structure, unemployment and parents’ education as covariates.

Statistically significant (p < 0.05) are in **bold**.

**Table 4:** Severe depression (odds ratios; 95% CI) by study year, sex, and parents’ unemployment and education

	**2000–2001**	**2002–2003**	**2004–2005**	**2006–2007**	**2008–2009**	**2010–2011**
**Parents’ education and employment**	**OR (95% CI)**	**OR (95% CI)**	**OR (95% CI)**	**OR (95% CI)**	**OR (95% CI)**	**OR (95% CI)**
**Boys**						
High education employed	1.00	1.00	1.00	1.00	1.00	1.00
High education unemployed	1.90 (1.57-2.30)	2.45 (2.03-2.96)	2.02 (1.69-2.41)	2.93 (2.48-3,46)	2.31 (1.97-2.71)	2.01 (1.70-2.38)
Medium education employed	0.88 (0.72-1.08)	0.88 (0.71-1.09)	0.86 (0.70-1.05)	1.10 (0.91-1.33)	0.83 (0.67-1.03)	0.83 (0.67-1.02)
Medium education unemployed	1.32 (1.06-1.64)	1.75 (1.42-2.17)	1.89 (1.54-2.32)	2.76 (2.26-3.37)	2.29 (1.87-2.79)	1.55 (1.24-1.92)
Low education employed	1.36 (1.01-1.83)	2.23 (1.71-2.91)	1.81 (1.38-2.41)	1.83 (1.34-2.50)	2.86 (2.16-3.79)	1.84 (1.37-2.47)
Low education unemployed	4.49 (3.56-5.67)	8.06 (6.45-10.10)	6.88 (5.54-8.53)	12.50 (10.10-15.30)	11.70 (9.39-14.50)	9.93 (8.21-12.00)
N	47586	50774	53057	54315	54132	51116
**Girls**						
High education employed	1.00	1.00	1.00	1.00	1.00	1.00
High education unemployed	2.03 (1.73-2.38)	2.14 (1.83-2.51)	2.30 (2.02-2.62)	2.10 (1.84-2.40)	2.12 (1.87-2.39)	2.01 (1.78-2.26)
Medium education employed	0.88 (0.72-1.08)	0.88 (0.71-1.09)	0.86 (0.70-1.05)	1.10 (0.91-1.33)	0.83 (0.67-1.03)	0.83 (0.67-1.02)
Medium education unemployed	1.32 (1.06-1.64)	1.75 (1.42-2.17)	1.89 (1.54-2.32)	2.76 (2.26-3.37)	2.29 (1.87-2.79)	1.55 (1.24-1.92)
Low education employed	1.36 (1.01-1.83)	2.23 (1.71-2.91)	1.81 (1.38-2.41)	1.83 (1.34-2.50)	2.86 (2.16-3.79)	1.84 (1.37-2.47)
Low education unemployed	2.93 (2.37-3.62)	3.44 (2.74-4.31)	4.01 (3.27-4.91)	4.18 (3.37-5.18)	5.47 (4.44-6.74)	4.86 (4.11-5.74)
N	47049	49236	51713	54005	54035	51066

Reference group: employed parents with high education.

Analyses were performed separately for boys and girls for two reasons: first, because the risk for depression differs among adolescent girls and boys, and second, because the literature suggests that the rate of depression has increased only among girls [16]. Time effect was studied using time as a continuous covariate variable in the models. The software package SPSS 18.0 for Windows (SPSS Inc, Chicago, Illinois) was used for all statistical analyses.

## Results

### Baseline characteristics of the study population

Among the participants the proportion of males was 50.3%, that of females 49.7%. The percentage of 8th graders was 50.5% and that of 9th graders 49.5%. At the time of the surveys, the 8th graders were 14–15 years old and the 9th graders 15–16 years old. About a third of the students lived in a family where one or both parents were unemployed (Table 1). More than half of the students lived in a family where one or both parents had high education; this proportion increased over the years (Table 1).

### Changes in depression during the study period

Among girls the rate of severe depression was slightly higher at the beginning of the second decade in this century (2010) than at the beginning of the first decade (2000). Severe depression was reported by 4% of girls and 2.1% of boys in 2000–2001 and by 4.7% and 2.2% respectively in 2010–2011 (Table 1). In all socio-economic groups except the group of adolescents whose parents had only a basic school education and were unemployed the prevalence of depression among girls was higher than among boys (Table 2). The prevalence of severe depression was higher among both boys and girls whose parents had a low education level or were unemployed, than among boys and girls whose parents had a medium or high level of education or were employed (Table 2). Regardless of parents’ educational background the prevalence of mild and moderate depression were also higher among the boys and girls whose parents were unemployed than among the adolescents whose parents were employed (Table 2). When studying the entire period, the prevalence of severe depression peaked among girls in 2010–2011 and among boys in 2008–2009 (Table 1). The prevalence of severe depression increased especially among those girls and boys whose parents were unemployed and had only a basic school education (Table 2). Among boys and girls whose parents had a low education level and were unemployed, severe depression was reported by 6.5% and 6.4% respectively in 2000–2001 and by 12.8% and 11.4% respectively in 2010–2011 (Table 2).

The multinomial regression models (Table 3) show the odds ratios of mild, moderate and severe depression (OR) and their 95% confidence intervals (CI) according to the study year. Among girls the odds of severe depression were lower in 2000–2001 than in all the subsequent periods except 2002–2003 (Table 3). All categories of depression were included in the model, and the results of severe depression are presented in Table 4, mild depression in Table 5 and moderate depression in Table 6. Covariates in the model in Table 4, in Table 5 and in Table 6 were grade, family structure, unemployment in the family and parents’ education. Multinomial regression models (Table 4) show changes of the odds of severe depression (OR, 95% CI) among adolescents by their parents’ education and employment during the study. The reference group is employed parents with high education. The odds of severe depression increased among both boys and girls whose parents had low education and were unemployed. The relative odds of severe depression were higher for those with low-educated and unemployed parents than for those with high-educated and employed parents. These relative odds were greater for boys than for girls. There was also an increasing trend in mild and moderate depression among both boys and girls whose parents had low education and had unemployed during past year (Tables 5 and 6).

**Table 5:** Mild depression (odds ratios; 95% CI) by study year, sex, and parents’ unemployment and education

	**2000–2001**	**2002–2003**	**2004–2005**	**2006–2007**	**2008–2009**	**2010–2011**
**Parents’ education and employment**	**OR (95% CI)**	**OR (95% CI)**	**OR (95% CI)**	**OR (95% CI)**	**OR (95% CI)**	**OR (95% CI)**
**Boys**						
High education employed	1.00	1.00	1.00	1.00	1.00	1.00
High education unemployed	1.43 (1.29-1.59)	1.40 (1.26-1.55)	1.51 (1.38-1.66)	1.49 (1.36-1.64)	1.53 (1.40-1.67)	1.76 (1.59-1.96)
Medium education employed	0.98 (0.89-1.08)	0.95 (0.87-1.05)	1.06 (0.97-1.16)	1.05 (0.96-1.15)	1.09 (0.99-1.19)	0.99 (0.89-1.09)
Medium education unemployed	1.35 (1.21-1.50)	1.33 (1.19-1.48)	1.55 (1.39-1.72)	1.60 (1.43-1.79)	1.64 (1.47-1.83)	1.46 (1.30-1.63)
Low education employed	1.13 (0.97-1.32)	1.16 (0.98-1.36)	1.17 (0.99-1.39)	1.20 (1.02-1.42)	1.24 (1.02-1.50)	1.21 (0.98-1.50)
Low education unemployed	1.52 (1.28-1.82)	1.67 (1.38-2.03)	1.62 (1.34-1.96)	1.92 (1.57-2.35)	1.99 (1.61-2.47)	1.73 (1.44-2.09)
N	47586	50774	53057	54315	54132	51116
**Girls**						
High education employed	1.00	1.00	1.00	1.00	1.00	1.00
High education unemployed	1.22 (1.12-1.33)	1.36 (1.24-1.48)	1.35 (1.25-1.46)	1.47 (1.36-1.60)	1.44 (1.33-1.55)	1.48 (1.37-1.59)
Medium education employed	1.05 (0.97-1.12)	1.09 (1.01-1.17)	1.09 (1.02-1.17)	1.16 (1.09-1.23)	1.15 (1.08-1.23)	1.13 (1.05-1.22)
Medium education unemployed	1.36 (1.25-1.47)	1.49 (1.38-1.62)	1.52 (1.40-1.65)	1.55 1.42-1.68)	1.56 (1.43-1.69)	1.48 (1.35-1.61)
Low education employed	1.16 (1.03-1.31)	1.16 (1.02-1.32)	1.13 (0.96-1.29)	1.17 (1.02-1.33)	1.30 (1.12-1.52)	1.21 (1.06-1.38)
Low education unemployed	1.40 (1.22-1.60)	1.53 (1.32-1.78)	1.63 (1.40-1.90)	1.68 (1.43-1.99)	1.83 (1.54-2.18)	1.93 (1.69-2.20)
N	47049	49236	51713	54005	54035	51066

Reference group: employed parents with high education.

**Table 6:** Moderate depression (odds ratios; 95% CI) by study year, sex, and parents’ unemployment and education

	**2000–2001**	**2002–2003**	**2004–2005**	**2006–2007**	**2008–2009**	**2010–2011**
**Parents’ education and employment**	**OR (95% CI)**	**OR (95% CI)**	**OR (95% CI)**	**OR (95% CI)**	**OR (95% CI)**	**OR (95% CI)**
**Boys**						
High education employed	1.00	1.00	1.00	1.00	1.00	1.00
High education unemployed	1.50 (1.34-1.68)	1.61 (1.43-1.81)	1.81 (1.63-2.01)	2.10 (1.90-2.32)	1.92 (1.74-2.11)	1.76 (1.59-1.96)
Medium education employed	0.84 (0.75-0.94)	0.96 (0.86-1.08)	1.03 (0.93-1.15)	1.12 (1.01-1.24)	1.10 (0.99-1.22)	0.93 (0.83-1.05)
Medium education unemployed	1.34 (1.19-1.51)	1.56 (1.38-1.76)	1.54 (1.36-1.74)	1.88 (1.66-2.14)	1.59 (1.40-1.81)	1.73 (1.53-1.96)
Low education employed	0.99 (0.82-1.19)	1.22 (1.01-1.47)	1.19 (0.98-1.44)	1.58 (1.32-1.89)	1.66 (1.36-2.03)	1.21 (0.98-1.50)
Low education unemployed	1.50 (1.24-1.83)	2.61 (2.15-3.16)	1.73 (1.39-2.14)	3.13 (2.57-3.81)	2.81 (2.26-3.49)	1.92 (1.55-2.37)
N	47586	50774	53057	54315	54132	51116
**Girls**						
High education employed	1.00	1.00	1.00	1.00	1.00	1.00
High education unemployed	1.58 (1.45-1.74)	1.66 (1.51-1.82)	1.57 (1.45-1.71)	1.92 (1.77-2.07)	1.68 (1.56-1.81)	1.68 (1.56-1.81)
Medium education employed	1.19 (1.11-1.29)	1.25 (1.16-1.35)	1.15 (1.07-1.23)	1.23 (1.15-1.31)	1.22 (1.14-1.31)	1.20 (1.11-1.29)
Medium education unemployed	1.82 (1.67-1.97)	1.83 (1.68-1.99)	1.87 (1.73-2.04)	1.99 (1.83-2.17)	2.00 (1.84-2.17)	1.94 (1.78-2.11)
Low education employed	1.44 (1.27-1.63)	1.44 (1.26-1.65)	1.46 (1.28-1.67)	1.42 (1.24-1.62)	1.60 (1.38-1.87)	1.50 (1.32-1.70)
Low education unemployed	2.04 (1.78-2.32)	2.31 (2.00-2.68)	2.28 (1.98-2.64)	2.15 (1.82-2.53)	2.26 (1.90-2.69)	2.64 (2.32-3.00)
N	47049	49236	51713	54005	54035	51066

Reference group: employed parents with high education.

## Discussion

The large dataset allowed the examination of depression trends in different socio-economic groups both among boys and girls. A novel finding of the present study is that there was a clear increasing trend in depression over time among both boys and girls who were socio-economically disadvantaged. While already high, the prevalence of depression among adolescents whose parents had a low education level and were unemployed nearly doubled during the study period and was ten times higher among the boys and four times higher among the girls, compared to boys and girls whose parents had a medium or high level of education and were employed.

Otherwise, our study showed overall rather limited changes in adolescent depression during the 12-year period. Among girls, the rate of severe depression was slightly higher in the beginning of the second decade of this century (2010) than in the beginning of the first decade (2000). Among boys the rate of depression varied. In all socio-economic groups, except for the group of adolescents whose parents had low education and were unemployed, the prevalence of depression among girls was higher, in some groups over two times higher than among boys.

The increasing trend in severe depression among the socio-economically disadvantaged youth is consistent with the findings of Sigfusdottir et al. [16] (2008) from Iceland and Sourander et al. [18] from Finland, but differs from the findings of the meta-analysis by Costello et al. [11]. Those studies, however did not explore the impact of socio-economic background, only simple trends.

Adolescent depression is associated with socio-economic disadvantage [18,19]. It has been suggested that the effects of poor living standards on depressive disorders are indirect and may be due to inequalities in living standards between population groups: that is, relative differences may be more important than absolute standards of living [33]. Although relative poverty is low and child poverty is rare in Finland compared with other EU countries [22], income inequality has increased in Finland in 1995–2005 even faster than in other Organisation for Economic Cooperation and Development (OECD) countries, and poverty among families has become more widespread [21]. Our results suggest that economic inequality is associated with increasing mental health inequalities. Thus, an increased rate of depression among disadvantaged adolescents may be explained by an increased relative poverty and income inequality. It is possible that socioeconomic disadvantage may be a more powerful risk factor for boys than for girls. Unfortunately, to our knowledge, this has not been studied, and further research is needed to confirm this assumption.

The goal, and the most difficult challenge of health policy, is to reduce inequality gaps. Our results suggest that mental health inequalities have increased among adolescents in Finland during the first decade of the 2000s. Targeting preventive efforts at disadvantaged adolescents and improving the living conditions of families might be useful in reducing the burden of depression. Research on planning measures to diminish mental health inequalities is desperately needed, and information on trends in mental health inequalities provides a good foundation for this research.

### Methodological considerations

This study was based on a nationwide population-based time-trend dataset study with a high participation rate, resulting in a large sample of 14–16-year-old Finnish adolescents. Only a few studies have included large enough samples to examine changes in small population groups. The sample of this age group is comprehensive because in Finland all those under 16 years of age must attend school, and in practice all children and adolescents (>99%) do so. Our data included the 535 schools that participated in every single survey, and 618,048 (94,635–108,320 biennially) pupils were present at these schools on the day of the survey and returned the questionnaire. The measurement of depression, timing of the study, and sampling were similar in each study year, and the surveys were conducted with the same method in the same schools over the entire study. Thus, the surveys are as comparable as possible. Even if there would be a bias in the measurement of depression, it was similar in all years and the time trends are still reliable.

Our findings are based on self-reported depression and cannot be explained by an improved identification of depression in health services or an increased willingness to seek treatment. Then again, if young people are now more likely to identify themselves as displaying the various symptoms of depression than previously, it may have an effect on their responses. However, the fact that this increase was seen largely among disadvantaged adolescents suggests that such a phenomenon may not be the only explanation.

This study has the limitations in reliability and accuracy that are inherent to self-reported data on depression. The BDI measures the respondent’s own perception of his or her depressive symptoms, but does not yield diagnostic data on depressive disorders. Severe depressive symptoms in adolescents are likely to be relatively persistent [35], and most of the morbidity associated with depression comes from the large numbers of people with depressive symptoms, rather than from the small number of cases with depressive disorders [36]. Furthermore, according to Lewinsohn et al. [2] symptoms of depression are usually better predictors of a depressive disorder than other risk factors.

Subjects (<0.7%) with incomplete responses on the depression rating scale were excluded from the analyses and we do not know if depression was more common among these adolescents than among those whose responses were complete. Among adolescents absent from school on the day of the survey, depressive symptoms may be more common. It is therefore possible that inclusion of these individuals would have resulted in a somewhat higher prevalence. The schools have been the same during the study period and there is no reason to assume that there would be a lot of change annually in school absences.

## Conclusion

Depression is increasing among adolescents with a socio-economically disadvantaged background. This is an indicator of increasing mental health disparities. Research on planning measures to diminish mental health inequalities is needed and information on trends in mental health inequalities provides a good foundation for this research.

## Competing interests

The authors declare that they have no competing interests.

## Authors’ contributions

AT, RK-H, AR and MM designed the study and wrote the protocol. AT wrote the first draft of the manuscript and managed the literature searches and analyses. TL undertook the statistical analysis. All authors participated in the writing of the report. All authors contributed to and have approved the final manuscript. All authors read and approved the final manuscript.

## Pre-publication history

The pre-publication history for this paper can be accessed here:

http://www.biomedcentral.com/1471-2458/14/408/prepub
